# Accuracy Difference of Noninvasive Blood Pressure Measurements by Sex and Height

**DOI:** 10.1001/jamanetworkopen.2022.15513

**Published:** 2022-06-07

**Authors:** Yasmine Abbaoui, Catherine Fortier, Louis-Charles Desbiens, Cédric Kowalski, Florence Lamarche, Annie-Claire Nadeau-Fredette, François Madore, Mohsen Agharazii, Rémi Goupil

**Affiliations:** 1 Hôpital du Sacré-Cœur de Montréal, Université de Montréal, Montréal, Quebec, Canada; 2 Hôpital Maisonneuve-Rosemont, Université de Montréal, Montréal, Quebec, Canada; 3 CRCHU de Québec-Université Laval, L’Hôtel-Dieu de Québec, Québec, Quebec, Canada

## Abstract

**Importance:**

Women are at higher risk of cardiovascular events than men with similar blood pressure (BP). Whether this discrepancy in risk is associated with the accuracy of brachial cuff BP measurements is unknown.

**Objectives:**

To examine the difference in brachial cuff BP accuracy in men and women compared with invasively measured aortic BP and to evaluate whether noninvasive central BP estimation varies with sex.

**Design, Setting, and Participants:**

This cross-sectional study enrolled 500 participants without severe aortic stenosis or atrial fibrillation from January 1 to December 31, 2019, who were undergoing nonurgent coronary angiography at a tertiary care academic hospital.

**Exposures:**

Simultaneous measurements of invasive aortic BP and noninvasive BP.

**Main Outcomes and Measures:**

Sex differences in accuracy were determined by calculating the mean difference between the noninvasive measurements (brachial and noninvasive central BP) and the invasive aortic BP (reference). Linear regression and mediation analyses were performed to identify mediators between sex and brachial cuff accuracy.

**Results:**

This study included 500 participants (145 female [29%] and 355 male [71%]; 471 [94%] White; mean [SD] age, 66 [10] years). Baseline characteristics were similar for both sexes apart from body habitus. Despite similar brachial cuff systolic BP (SBP) (mean [SD], 124.5 [17.7] mm Hg in women vs 124.4 [16.4] in men; *P* = .97), invasive aortic SBP was higher in women (mean [SD], 130.9 [21.7] in women vs 124.7 [20.1] mm Hg in men; *P* < .001). The brachial cuff was relatively accurate compared with invasive aortic SBP estimation in men (mean [SD] difference, −0.3 [11.7] mm Hg) but not in women (mean [SD] difference, −6.5 [12.1] mm Hg). Noninvasive central SBP (calibrated for mean and diastolic BP) was more accurate in women (mean [SD] difference, 0.6 [15.3] mm Hg) than in men (mean [SD] difference, 8.3 [14.2] mm Hg). This association of sex with accuracy was mostly mediated by height (3.4 mm Hg; 95% CI, 1.1-5.6 mm Hg; 55% mediation).

**Conclusions and Relevance:**

In this cross-sectional study, women had higher true aortic SBP than men with similar brachial cuff SBP, an association that was mostly mediated by a shorter stature. This difference in BP measurement may lead to unrecognized undertreatment of women and could partly explain why women are at greater risk for cardiovascular diseases for a given brachial cuff BP than men. These findings may justify the need to study sex-specific BP targets or integration of sex-specific parameters in BP estimation algorithms.

## Key Points

**Question:**

Do noninvasive brachial cuff blood pressure (BP) measurements accurately estimate the invasive aortic (or true) BP in men and women?

**Findings:**

In this cross-sectional study of 500 patients undergoing cardiac catheterization at a tertiary care academic hospital, women had significantly higher invasively measured aortic systolic BP (SBP) compared with men with similar brachial cuff SBP. This disparity was mostly explained by a difference in height, with shorter height associated with greater underestimation of the invasive aortic SBP by the brachial cuff.

**Meaning:**

These findings suggest that brachial cuff BP may significantly underestimate true aortic SBP in women, which may lead to unrecognized undertreatment of women compared with men and could partly explain why women are at higher risk of cardiovascular diseases for a given brachial cuff BP.

## Teaser

This cross-sectional study of patients undergoing cardiac catheterization assesses the association of sex with the ability of noninvasive blood pressure measurements to accurately estimate invasive aortic blood pressure.

## Introduction

The prevalence of cardiovascular disease is higher in men than women in most age groups, whereas the overall lifetime risk is similar for both sexes. This paradox can be explained by differences in longevity, cardiovascular disease risk factors, comorbid conditions, and treatments in men and women.^1,2^ Whether the association between hypertension and cardiovascular disease is different for each sex is controversial. In this regard, 2 studies^3,4^ have found a higher cardiovascular risk in women vs men with similar baseline blood pressure (BP). In addition, previous data suggest a possible association between greater underestimation of invasively measured brachial systolic BP (SBP) by the brachial cuff in women, suggesting that the accuracy of the brachial cuff compared with invasive aortic BP could also be influenced by biological sex.^5^ Whether this association is attributable to differences in morphologic factors, vascular phenotypes, hormonal changes, artery diameter, or other factors associated with the oscillometry procedure remains to be determined.

The Italian physicist and internist Scipione Riva-Rocci designed brachial cuff sphygmomanometry to provide a convenient surrogate of the aortic BP (also called true central BP or invasive central BP).^6^ A meta-analysis using invasively measured aortic BP values as reference has shown that the brachial cuff estimates aortic SBP with relatively good accuracy on average but with a high degree of variability.^7^ Thus, although it can accurately estimate the mean aortic BP on a populational level, the brachial cuff may lack accuracy on an individual level. Highly accurate BP measures are needed because even small BP variations can significantly affect the cardiovascular risk estimation and result in misclassification of a large portion of a population.^8^ This misclassification can lead to suboptimal drug therapy caused by overtreatment and medication-related adverse effects or undertreatment and failure to adequately prevent cardiovascular complications.^5,9,10^ This issue raises the importance of using accurate noninvasive BP to properly assess aortic BP. Noninvasive central BP devices have the potential to better estimate cardiovascular risk than brachial cuff BP because they aim to provide a more accurate measure of the invasive aortic BP and target organ perfusion.^7,11,12,13,14,15,16,17,18,19^

Overall, the association between sex and both brachial cuff accuracy and noninvasive central BP estimation remains unclear. As such, our primary aim was to investigate whether sex is associated with the accuracy of noninvasive BP measurements with regard to the invasively measured aortic BP. The secondary objectives were to characterize the determinants of brachial cuff accuracy and to quantify their association with sex.

## Methods

### Study Population

The invasive BP study cohort was composed of patients who had a clinical indication for coronary angiography at the Hôpital du Sacré-Coeur de Montréal in Quebec, Canada, from January 1 to December 31, 2019.^5^ All included participants (n = 500) were without severe aortic stenosis or atrial fibrillation. Invasive aortic BP and cuff-based noninvasive brachial and central BP measurements were recorded simultaneously in all participants. When feasible, invasive brachial artery BP was also measured. Reasons for nonmeasurement of invasive brachial BP were related to the procedure (femoral access or safety and time constraints). Race was self-reported by participant and was included to assess generalizability of the findings. Of those patients, we selected a subgroup without significant interarm SBP difference (>5 mm Hg, measured sequentially). This study followed the ARTERY Society task force consensus statement on protocol standardization.^20^ The Comité d'éthique de la recherche du CIUSSS du Nord-de-l’Île-de-Montréal approved the study, which adhered to the Declaration of Helsinki.^21^ Written informed consent was obtained from participants before study inclusion. This study followed the Strengthening the Reporting of Observational Studies in Epidemiology (STROBE) reporting guideline.^22^

### Invasive and Noninvasive BP Measures

See the eMethods in the Supplement for the detailed study protocol. At the end of the coronary angiography procedure, intra-arterial BPs were measured in the ascending aorta (invasive aortic BP). When possible, additional recordings were taken after pulling back the catheter midhumerus to the brachial artery (invasive brachial BP). An automatic BP monitor (Mobil-O-Graph NG, I.E.M. GmbH)^23,24^ was used for all noninvasive brachial and central BP readings. Through pulse wave analysis, this device first measures brachial cuff BP then reinflates to capture the pulse waveform and derive noninvasive central BP, simultaneously with type I (SBP and diastolic BP [DBP]) and type II (mean arterial pressure [MAP] and DBP) calibrations. Recordings of noninvasive BPs were made simultaneously to the invasive aortic measurement, timed to coincide with the cuff deflation.

### Statistical Analysis

Statistical analyses were conducted between July 2021 and April 2022. Normally distributed continuous data are presented as mean (SD) and compared with 2-tailed, unpaired *t* tests or 1-way analysis of variance with Bonferroni corrections for post hoc analyses, as appropriate. Categorical data were compared with Pearson χ^2^ tests. Two-sided *P* < .05 was considered significant. Analyses were performed with SPSS Statistics, version 25.0 (IBM Inc). The accuracy of noninvasive BPs (brachial, type I, and type II central) to estimate invasive BPs (aortic and brachial) was determined by calculating the mean (SD) difference between these 2 measurements.^20^ In accordance with the ARTERY Society consensus,^20^ an accurate device demands a mean difference (accuracy) of 5 mm Hg or less with an SD (precision) of 8 mm Hg or less compared with the reference invasive measure. Each noninvasive SBP measure is also compared with the corresponding invasive aortic SBP in modified Bland-Altman plots.^20^ The true aortic to brachial SBP amplification was calculated by subtracting the invasive aortic SBP from the invasive brachial SBP (if available). All values were compared between sexes.

To identify factors associated with the accuracy of the brachial cuff SBP compared with the invasive aortic SBP, we used linear regression models. We included in a model all clinical characteristics plausibly associated with BP accuracy (sex, age, height, weight, brachial cuff DBP, brachial cuff pulse pressure, heart rate, augmentation index at 75 beats/min, aortic pulse wave velocity, estimated glomerular filtration rate, active smoking status, type 2 diabetes, presence of ≥1 diseased vessel on coronary angiogram, and use of antihypertensive drugs, aspirin, and statins). In parallel, we used a least absolute shrinkage and selection operator (LASSO) regression to select variables that would most improve the accuracy and avoid overfitting in the model.

To quantify the influence of the identified factors on accuracy in the association between sex and accuracy, a mediation analysis was performed. Mediation analyses are used to test how given mediators (factors associated with accuracy) influence the association between an independent variable (sex) and an outcome variable (brachial cuff accuracy). These analyses allow the quantification of the total effect (the association between sex and brachial cuff accuracy), the direct effect (the total effect without the influence of mediators), and the indirect effect (the effect of sex on accuracy attributable to mediators). The percent mediation represents the proportion of the total effect attributable to each mediator. Because the degree of underestimation of the intra-arterial brachial SBP by the brachial cuff likely influences the association between sex and brachial cuff accuracy,^5^ we repeated the mediation analysis but with the addition of the difference between brachial cuff and intra-arterial brachial SBP as a mediator in the subgroup of participants with available intra-arterial brachial BP. All mediation analyses were performed using the PROCESS Statistical Package for SPSS, release 3.5.3, with bootstrapping with 5000 resamples to calculate indirect effect and 95% CIs.^25^

A sensitivity analysis was performed to assess whether significant interarm BP differences could influence our findings because exclusion of such individuals may preclude selecting patients with significant upper arm stenotic atherosclerotic disease, which could falsely lower the noninvasive BP in the affected arm. As such, the accuracy of all noninvasive BPs compared with the intra-arterial aortic BP was recalculated in the subgroup of participants with intra-arterial brachial BP measurements in which all individuals with significant interarm SBP differences (>5 mm Hg) were excluded. Other sensitivity analyses were performed to assess whether procedural factors could have influenced our results. As such, the accuracies of the brachial cuff BP compared with the invasive aortic BP according to sex were compared according to (1) the use of 5F or 6F catheters, (2) the administration of any vasoactive drugs within 5 minutes of the invasive BP measurements, (3) whether the primary study cardiologist (C.K.) performed the measurements, and (4) the signal quality of the automatic BP monitor. Finally, sensitivity analyses were performed to examine the association with other body size parameters by replacing weight with body mass index (calculated as weight in kilograms divided by height in meters squared) or body surface area (Du Bois formula^26^).

## Results

The invasive BP study cohort consists of 500 participants (145 female [29%] and 355 male [71%]; 471 [94%] White; mean [SD] age, 66 [10] years) with invasive aortic BP recordings, of whom 303 had invasive brachial BP measurements without significant interarm BP difference (eFigure 1 in the Supplement). Women were shorter, weighed less, and had had less coronary artery disease than men but had similar age, body mass index, and comorbidities (Table 1). Both sexes had highly similar brachial cuff SBP (mean [SD], 124.4 [16.4] mm Hg in men and 124.5 [17.7] in women; *P* = .97), but women had lower brachial cuff DBP and thus higher brachial cuff pulse pressure.

**Table 1.** Clinical Characteristics of the Study Participants^a^

Clinical characteristic	Women (n = 145)	Men (n = 355)	*P* value^b^
Age, mean (SD), y	66.1 (10.8)	65.6 (9.6)	.61
Height, mean (SD), cm	159.3 (6.8)	174.4 (7.0)	<.001
Weight, mean (SD), kg	73.5 (17.5)	86.4 (17.9)	<.001
BMI, mean (SD)	28.9 (6.4)	28.4 (5.3)	.33
Race			
Black	2 (1.4)	14 (3.9)	.11
White	134 (92)	336 (95)	
Other^c^	8 (5.5)	5 (1.4)	
Active smoking	38 (26)	86 (24)	.64
Diabetes	38 (26)	103 (29)	.53
eGFR, mean (SD), mL/min/1.73 m^2^^d^	79.3 (18.3)	80.6 (17.0)	.45
≥1 Diseased vessel	75 (52)	257 (72)	<.001
Antihypertensive medication	110 (76)	289 (81)	.16
Aspirin use	104 (72)	250 (70)	.77
Statin use	97 (67)	251 (71)	.40
Brachial cuff SBP, mean (SD), mm Hg	124.5 (17.7)	124.4 (16.4)	.97
Brachial cuff pressure, mean (SD), mm Hg			
DBP	73.5 (10.7)	77.9 (10.5)	<.001
MAP	97.0 (13.0)	99.1 (12.2)	.08
PP	51.0 (12.8)	46.5 (11.2)	<.001
Heart rate, mean (SD), beats/min	71.1 (13.2)	66.1 (11.3)	<.001
Aortic pulse wave velocity, mean (SD), m/s	9.4 (2.1)	9.4 (1.7)	.93
Augmentation index at 75 beats/min, mean (SD)	26.0 (13.4)	17.1 (13.1)	<.001
Reflection magnitude, mean (SD)	66.4 (9.0)	67.1 (9.2)	.42

Abbreviations: BMI, body mass index (calculated as weight in kilograms divided by height in meters squared); DBP, diastolic blood pressure; eGFR, estimated glomerular filtration rate; MAP, mean arterial pressure; PP, pulse pressure; SBP, systolic blood pressure.

^a^ Data are presented as number (percentage) of patients unless otherwise indicated.

^b^ *P* values were calculated using the Pearson χ^2^ and *t* tests.

^c^ Other race was a self-reported designation.

^d^ Estimated using the Chronic Kidney Disease Epidemiology Collaboration formula.

Table 2 gives comparisons of invasive aortic BP and noninvasive BPs between women and men. The invasive aortic SBP was significantly higher in women compared with men (mean [SD], 130.9 [21.7] vs 124.7 [20.1] mm Hg; difference, 6.2 mm Hg; *P* < .001) despite the highly similar brachial cuff SBPs. Regarding DBP, invasive aortic values were similar between women and men (mean [SD], 68.7 [13.4] vs 68.7 [10.1] mm Hg; *P* > .99), but women had lower brachial cuff DBP values than men (mean [SD], 73.5 [10.7] vs 77.9 [10.5] mm Hg; *P* < .001). Invasive aortic and noninvasive brachial cuff MAPs were similar in men and women. In the subgroup of patients for whom invasive brachial BP was available, the invasive brachial SBP was also higher in women than men (mean [SD], 136.2 [20.8] vs 130.8 [20.3] mm Hg; *P* = .04), whereas invasive brachial DBP did not differ between the sexes (eTable 1 in the Supplement). Invasive aortic to brachial SBP amplification was similar between women and men (mean [SD], 6.3 [10.3] vs 6.8 [9.1] mm Hg; *P* = .65).

**Table 2.** Comparison of Invasive and Noninvasive Blood Pressures in the Study Participants^a^

Component	Women (n = 145)	Men (n = 355)	*P* value
SBP, mm Hg			
Invasive aortic	130.9 (21.7)	124.7 (20.1)	<.001
Brachial cuff	124.5 (17.7)	124.4 (16.4)	.97
Type I noninvasive central	113.6 (16.7)	116.1 (15.9)	.13
Type II noninvasive central	131.5 (18.6)	133.2 (17.9)	.34
Difference from invasive aortic SBP, mm Hg			
Brachial cuff	–6.5 (12.1)	–0.3 (11.7)	<.001
Type I noninvasive central	–17.3 (13.1)	–8.8 (13.1)	<.001
Type II noninvasive central	0.6 (15.3)	8.3 (14.2)	<.001
DBP, mm Hg			
Invasive aortic	68.7 (13.4)	68.7 (10.1)	>.99
Brachial cuff	73.5 (10.7)	77.9 (10.5)	<.001
Type I noninvasive central	74.9 (10.7)	79.1 (10.7)	<.001
Type II noninvasive central	75.7 (10.7)	79.7 (10.6)	<.001
Difference from invasive aortic DBP, mm Hg			
Brachial cuff	4.9 (12.2)	9.2 (8.3)	<.001
Type I noninvasive central	6.2 (12.2)	10.5 (8.4)	<.001
Type II noninvasive central	7.0 (12.8)	11.1 (8.7)	.001
MAP, mm Hg			
Invasive aortic	93.8 (13.8)	91.4 (12.2)	.06
Brachial cuff	97.0 (13.0)	99.1 (12.2)	.08
Difference from invasive aortic MAP, mm Hg			
Brachial cuff	3.1 (8.0)	7.7 (7.7)	<.001

Abbreviations: DBP, diastolic blood pressure; MAP, mean arterial pressure; SBP, systolic blood pressure.

^a^ Mean (SD) differences represent the difference between noninvasive blood pressure and intra-arterial invasive aortic blood pressure measurements. Type I noninvasive central blood pressure was obtained through calibration with brachial cuff SBP and DBP. Type II noninvasive central blood pressure was obtained through calibration with brachial cuff mean blood pressure and DBP.

### Accuracy of Noninvasive BP According to Sex

**Figure 1.**
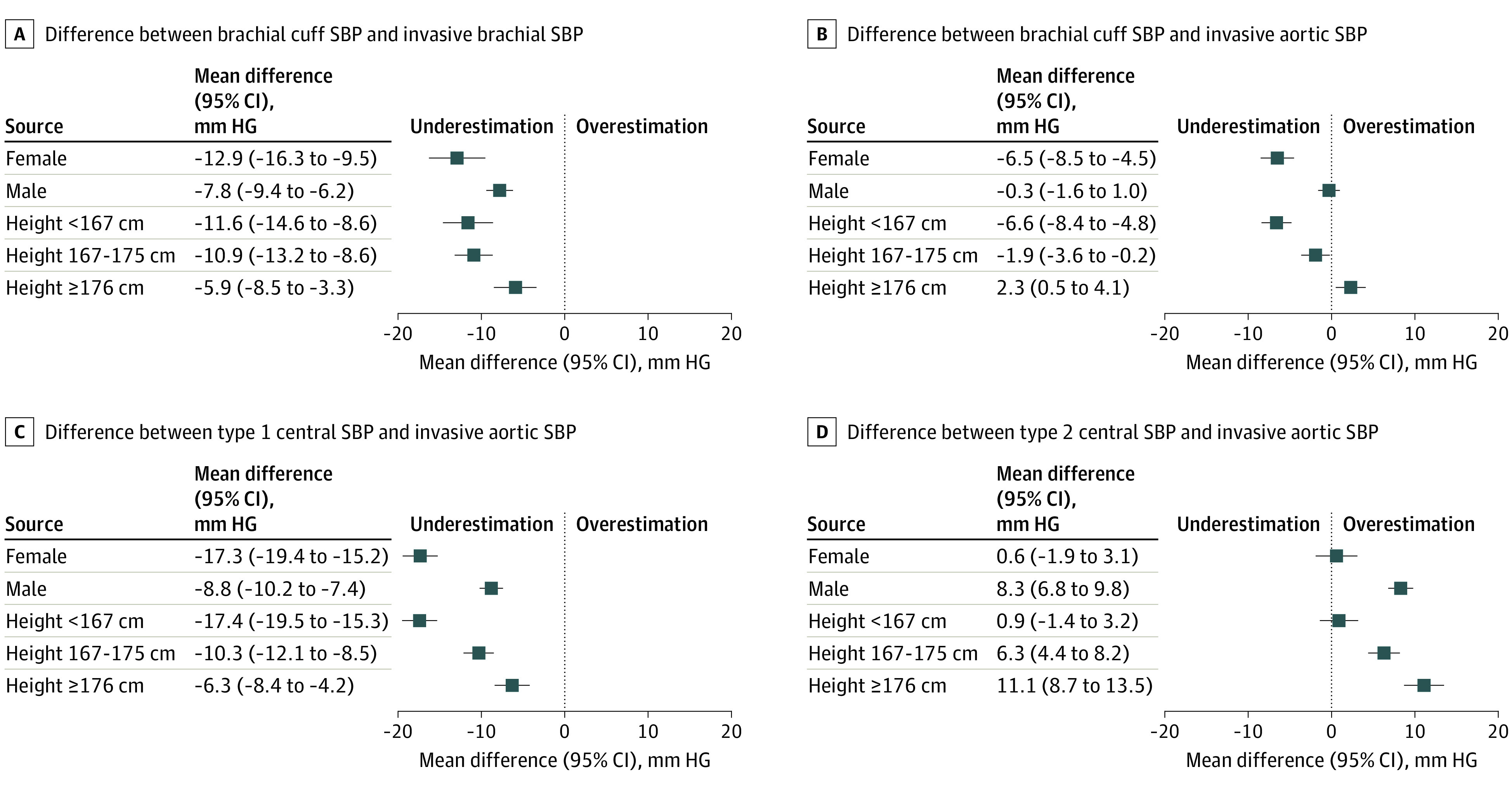
Mean Differences Between Invasive (Brachial and Aortic) and Noninvasive (Brachial Cuff, Type I Noninvasive Central, and Type II Noninvasive Central) Systolic Blood Pressure (SBP) According to Sex and Tertiles of Height Error bars indicate 95% CIs. Dotted line represents the point where neither overestimation (>0) nor underestimation (<0) occurs (indicative of high accuracy).

In the overall cohort (irrespective of sex), the mean (SD) accuracies against the invasive aortic SBP were −2.1 (12.1) mm Hg for brachial cuff noninvasive central BP, −11.2 (13.6) mm Hg for type I noninvasive central BP, and 6.1 (14.9) mm Hg for type II noninvasive central BP. The brachial cuff was less accurate against invasive aortic SBP estimation in women compared with men (mean [SD], differences, −6.5 [12.1] for women and −0.3 [11.7] mm Hg for men) (Table 2). Type I noninvasive central BP greatly underestimated invasive aortic SBP in both sexes but to a higher degree in women (mean [SD] difference, −17.3 [13.1] mm Hg) than in men (mean [SD] difference, −8.8 [13.1] mm Hg; *P* < .001). However, type II noninvasive central BP was the most accurate noninvasive BP in women (although with low precision), with a mean (SD) difference of 0.6 (15.3) mm Hg as opposed to 8.3 (14.2) mm Hg for men. Figure 1 illustrates the accuracy with 95% CIs of all 3 types of noninvasive BP measures compared with invasive aortic SBP. eFigure 2 in the Supplement shows the Bland-Altman plots. This differing accuracy of the brachial cuff was similar in all sensitivity analyses assessing the potential role of procedural factors (eTable 2 in the Supplement). All 3 noninvasive DBPs overestimated the invasive aortic DBP to a similar degree, although to a greater degree in men (Table 2; eFigure 3 in the Supplement).

### Factors Associated With Brachial Cuff Accuracy

To determine factors associated with the difference between brachial cuff SBP and invasive aortic SBP, we first used a linear regression model that included sex and all available potential confounding factors. From this model, the only variables that proved to be independently associated with the brachial cuff SBP accuracy were sex, age, and height (eTable 3 in the Supplement). In parallel, we used a LASSO regression for variable selection, which identified only height as a factor associated with brachial cuff SBP accuracy. In sensitivity analyses, replacing weight with other body size parameters (body mass index and body surface area) did not alter the association of brachial cuff SBP accuracy with sex, age, and height (eTable 4 in the Supplement).

**Figure 2.**
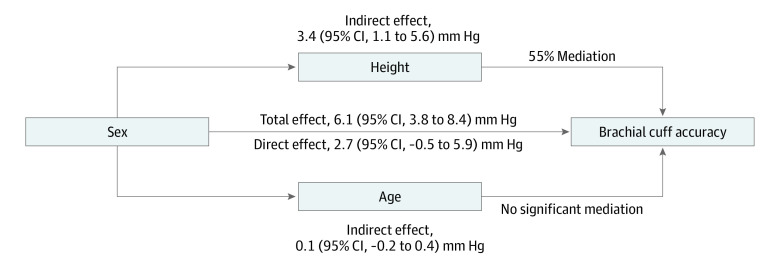
Mediation Effect by Height and Age in the Association Between Sex and Brachial Cuff Systolic Blood Pressure Accuracy Total effect represents the summation of the direct effect of sex on brachial cuff systolic blood pressure accuracy with the indirect effects from height and age. Effects are expressed as magnitudes of the difference between brachial cuff systolic blood pressure and invasive aortic systolic blood pressure. Percent mediation represents the ratio of the total effect attributable to the corresponding indirect effect.

**Figure 3.**
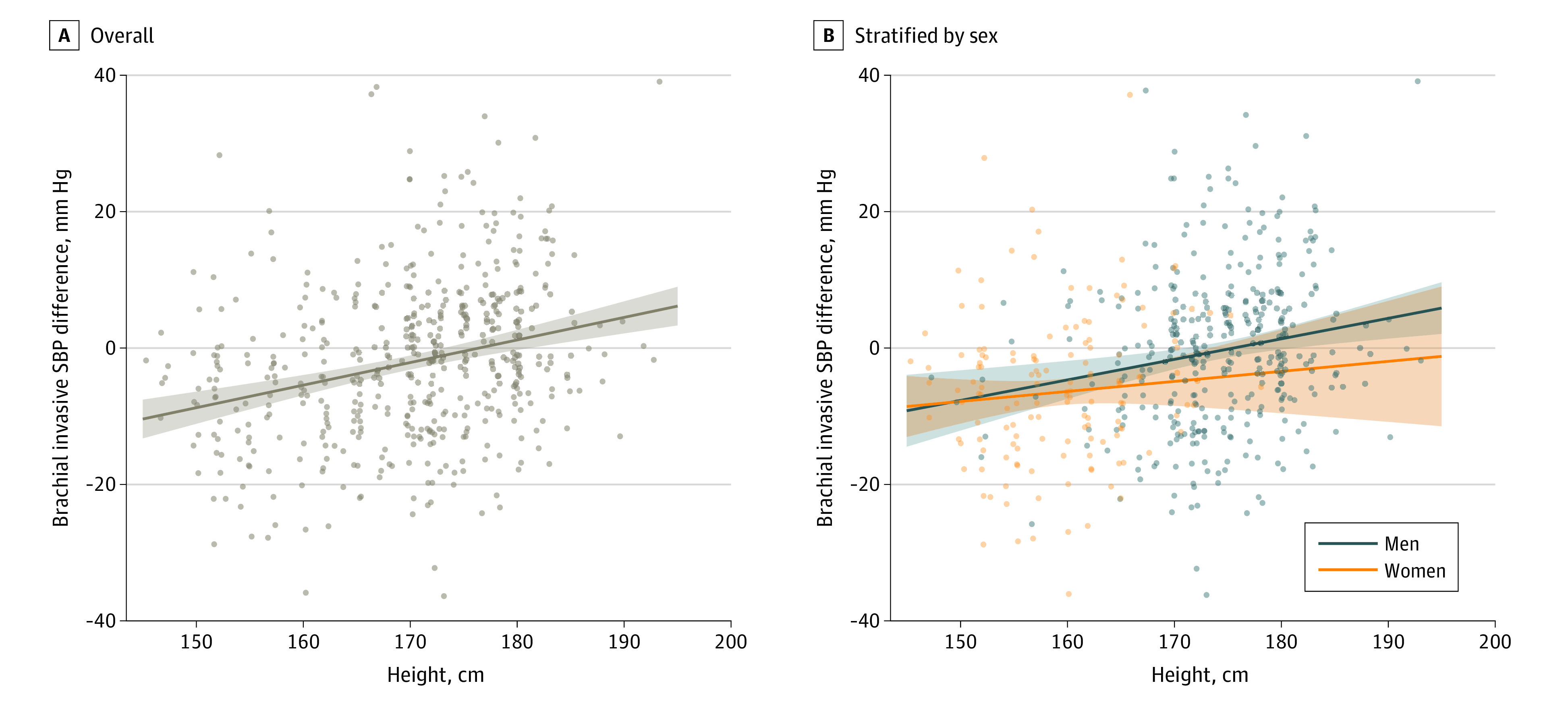
Brachial Cuff Systolic Blood Pressure (SBP) Accuracy According to Height for Overall Cohort and Stratified by Sex

To further analyze the association of height and age with accuracy, we evaluated the accuracy of all noninvasive BPs according to tertiles of height (<167, 167-175, and ≥175 cm) and decades of age. This analysis showed that height was associatd with noninvasive SBP accuracy, with participants of shorter stature having the greatest accuracy from type II noninvasive central SBP (mean [SD] difference vs invasive aortic SBP, 0.9 [14.8] mm Hg; *P* < .001) and the lowest from brachial cuff SBP (mean [SD] difference vs invasive brachial, −11.6 [14.3] mm Hg; *P* = .01) (Figure 2; eTable 5 in the Supplement). This finding was also evident in sex-stratified analyses that compared the accuracy of noninvasive BP measurements in each tertile of height (eTable 6 in the Supplement), although the finding was not statistically significant in women, possibly because of lower power. Figure 3 illustrates that the lesser underestimation of invasive aortic SBP from the brachial cuff seen with increasing height was present in both women and men. As with sex, the differences in DBP accuracy were not as compelling between tertiles of height (eFigure 3 in the Supplement). Regarding age, our data indicate a progressively greater underestimation of invasive aortic SBP with the brachial cuff and type I central measurements, more so in women (eTable 7 in the Supplement). The accuracy with type II central SBP appeared not to be associatd with age.

We conducted a mediation analysis to characterize the direct effect of sex on brachial cuff accuracy. Both height and age were included in this analysis because they were independently associated with brachial cuff accuracy in the first linear regression analysis. This mediation analysis indicates that most of the total effect of sex on brachial cuff accuracy (6.1 mm Hg; 95% CI, 3.8-8.4 mm Hg) was attributable to an indirect effect from height (3.4 mm Hg; 95% CI, 1.1-5.6 mm Hg; 55% mediation) (Figure 2). The remaining direct effect of sex was no longer statistically significant at 2.7 mm Hg (95% CI, –0.5 to 5.9 mm Hg) after removal of the height association. Age did not significantly mediate the association between sex and brachial cuff accuracy (0.1 mm Hg; 95% CI, –0.2 to 0.4 mm Hg). In addition, in the subgroup of participants with available intra-arterial brachial BP, we evaluated the direct effect of the difference between the brachial cuff SBP and the invasive brachial SBP. In this analysis, the addition of brachial cuff underestimation further reduced the residual direct effect of sex (0.9 mm Hg; 95% CI, –1.4 to 3.2 mm Hg) and partially attenuated the effect of height (2.1 mm Hg; 95% CI, 0.5-3.6 mm Hg) on accuracy.

## Discussion

In this cross-sectional study of invasive aortic BP measurements, we found that compared with men with similar brachial cuff systolic BP, women had unrecognized significantly higher invasive aortic SBP. This difference in the accuracy of brachial cuff BP devices between sexes was mostly mediated by height. In addition, only type II noninvasive central SBP appeared to provide accurate representation of the invasive aortic BP in women, whereas conventional brachial cuff SBP was the most accurate in men.

Cardiovascular disease and hypertension are more common in men and postmenopausal women than in premenopausal women.^3,19,27^ As a result, women with coronary heart disease are generally older than men and have more cardiovascular risk factors.^2,28^ In addition, women have a higher life expectancy than men, which results in a larger elderly population of women in whom cardiovascular disease prevalence is at its highest.^1^ Female hormones, mainly estrogen, could have protective effects before menopause.^19,29^ Furthermore, healthy lifestyle behaviors are more common in women than in men without coronary heart disease.^30^ For a similar age, men have a higher BP than women, but after menopause, the reverse phenomenon is observed.^3,31^ Although the decrease in levels of estrogens could play a role in women’s increase in BP after menopause, other factors are involved, because hormone replacement therapy poorly decreases BP.^31^ Some studies^3,32^ have found that among women and men with similar SBP women have a higher cardiovascular risk. Our findings suggest this increased risk could be caused by unrecognized higher invasive aortic BP in women compared with men with similar brachial cuff BP.

To our knowledge, the association between height and the accuracy of BP devices (assessed against invasive BP measurements) has never been investigated before this study. Indirect evidence solely based on noninvasive measurement suggests that sex, through differences in height, could mediate the association between central and peripheral BP and that arterial stiffness and wave reflections play a role in that association. As the forward pulse wave is reflected on arterial bifurcations and zones of varying impedance, it creates a backward wave that then amplifies the incident wave.^33^ Depending on height, forward and backward waves intersect at different locations in the arterial tree, which changes SBP amplification and could explain the difference in BP accuracy. Indeed, the reflected waves arrive earlier in the systole in shorter people, leading to lower SBP amplification.^34^ Stiffened arteries lead to higher pulse wave velocities, which cause backward waves to travel faster.^35^ However, our data do not support that the sex-based difference in brachial cuff accuracy is solely explained by central hemodynamics. In our study, invasively measured SBP amplification was similar in both sexes, when it would be expected to be lower in women and shorter people. In addition, estimated pulse wave velocity was comparable in both sexes. However, the Mobil-O-Graph algorithm to estimate pulse wave velocity may be less accurate because it is mainly based on age and brachial cuff BP,^36,37^ which are 2 variables that were similar in both sexes in our study. Evidently, differences in height alone do not explain the discrepancy in brachial cuff accuracy, as suggested by the 55% mediation. Brachial cuff underestimation of the true brachial SBP appears to explain most of the residual effect of sex on accuracy, although an explanation for this phenomenon remains undetermined. Factors related to vascular phenotype, blood vessel diameter, sensors sensitivity, or how the oscillometric wave is captured and interpreted by the device may be implicated. Age was also associated with brachial cuff accuracy, as previously described.^38^ However, it did not mediate the association between sex and brachial cuff accuracy once height was considered.

Noninvasive central BP is a better estimate of invasive aortic BP than brachial cuff BP and may be better in the assessment of cardiovascular outcomes, although this is possibly device dependent, with type I calibration unlikely to be clinically superior.^11,12,13,14,15,16,17,18,19,39^ Indeed, major organs get their blood flow from the aorta, not the brachial artery.^17^ It has been established that using type II calibration provides more accurate results than using type I calibration.^40,41,42^ Our study shows that in women, type II noninvasive central BP outperforms the brachial cuff BP, whereas in men, the brachial cuff provides the most accurate results. The explanation for this phenomenon appears to be straightforward. In men, the underestimation of the invasive brachial SBP by the brachial cuff was almost completely compensated by the aortic to brachial SBP amplification, resulting in a brachial cuff SBP similar to the invasive aortic SBP. However, in women, the underestimation of the invasive brachial SBP by the brachial cuff was twice greater, which means the similar level of SBP amplification fails to fully compensate, yielding an underestimation of the aortic SBP by the brachial cuff. The high accuracy of type II noninvasive central SBP in women could be merely because this type of noninvasive central BP yields values that are usually higher than the corresponding brachial cuff SBPs, therefore compensating for the underestimation of the invasive aortic SBP by the brachial cuff.

In women, of all the noninvasive BP methods, only type II noninvasive central BP approached the accuracy criteria set by the ARTERY Society, and in men, only brachial cuff BP did. However, all had high variability (SD), indicating low precision. It is crucial to have accurate devices in clinical practice because the smallest variation in estimated BP can have important effects on cardiovascular risk management.^8^ Inaccuracy can lead to misclassification of populations and, on an individual level, to undertreatment or overtreatment of patients and suboptimal drug therapy or adverse events.^5,9,10^

### Strengths and Limitations

This study has several strengths. To the best of our knowledge, our study is the first to characterize the association of sex with brachial cuff and noninvasive central BP accuracy. This study had a distinctively large sample size and used a protocol aligned with the ARTERY Society recommendations.^20^ Nonetheless, this study has some limitations. First, the fact that all patients had clinical indications to undergo coronary angiography limits the generalizability of our findings, although it may not be ethically possible to perform this study in other populations. Second, patients were almost exclusively White. Third, noninvasive BP measurements had to be timed to be recorded at the same time as invasive aortic measurements, but this was not always possible because of technical issues. Fourth, it could be argued that sex-specific procedural factors (such as a greater propensity for use of 5F catheters in women or greater use of vasoactive drugs because of vasospasms of lower-caliber arteries) may have contributed to our findings, although this possibility is not supported by our various sensitivity analyses. Fifth, we cannot exclude the presence of residual confounding. Sixth, our noninvasive central BP findings could be specific to the Mobil-O-Graph device. However, our data are concordant with findings on the association of age and brachial cuff accuracy from the Invasive Blood Pressure Consortium, which pooled individual-level data from 31 studies and 22 different cuff BP devices, suggesting data from the Mobil-o-Graph could be generalizable to other devices.^38^ Nonetheless, all devices should be tested for reliable brachial cuff measurements to adequately measure noninvasive central BP.

## Conclusions

In this cross-sectional study, although conventional brachial cuff BP adequately estimated aortic BP in men, it lacked accuracy in women mostly because of differences in height. This finding could have considerable consequences for clinical practice because, for a similar brachial cuff SBP, women have higher aortic SBP, which could explain why they are at higher risk of cardiovascular disease than men. Type II noninvasive central BP appears to be the best method to estimate aortic SBP in women and may need to be integrated in clinical practice. To obtain accurate noninvasive BP measurements, novel algorithms will need to integrate clinical characteristics known to influence accuracy, such as age, sex, and height. Although conventional brachial cuff BP has proven its utility over the years, this study paves the way toward a more individualized approach of assessing BP.

## Notes

Supplement. **eMethods.** Supplemental Methods **eTable 1.** Comparison of Invasive and Non-Invasive Blood Pressures in Females and Males in the Subset of Participants With Available Invasive Brachial Blood Pressure **eTable 2.** Difference Between Invasive Central Aortic SBP and Non-Invasive Brachial Cuff SBP According to Sex and Various Procedural Factors **eTable 3.** Determinants of the Difference Between Brachial Cuff and Invasive Aortic Systolic Blood Pressures **eTable 4.** Determinants of the Difference Between Brachial Cuff and Invasive Aortic Systolic Blood Pressures (Sensitivity Analyses With Body Mass Index or Body Surface Area Instead of Weight) **eTable 5.** Comparison of Invasive and Non-Invasive Blood Pressures According to Tertiles of Height **eTable 6.** Comparison of Invasive and Non-Invasive Blood Pressures According to Tertile of Height and Sex **eTable 7.** Comparison of Invasive and Non-Invasive Blood Pressures According to Age Groups and Sex **eFigure 1.** Study Flowchart **eFigure 2.** Modified Bland-Altman Plots According to Sex and Type of Non-Invasive Systolic Blood Pressure Measurement **eFigure 3.** Mean Differences Between Invasive (Brachial and Aortic) and Non-Invasive (Brachial Cuff, Type I Central and Type II Central) Diastolic Blood Pressure According to Sex and Tertiles of Height
