# Supplementary material for: Accuracy Difference of Noninvasive Blood Pressure Measurements by Sex and Height
Source: JAMA Netw Open. 2022 Jun 7;5(6):e2215513. doi: 10.1001/jamanetworkopen.2022.15513 (PMC9175075; doi:10.1001/jamanetworkopen.2022.15513)
Supplement: Supplement. — eMethods. Supplemental Methods eTable 1. Comparison of Invasive and Non-Invasive Blood Pressures in Females and Males in the Subset of Participants With Available Invasive Brachial Blood Pressure eTable 2. Difference Between Invasive Central Aortic SBP and Non-Invasive Brachial Cuff SBP According to Sex and Various Procedural Factors eTable 3. Determinants of the Difference Between Brachial Cuff and Invasive Aortic Systolic Blood Pressures eTable 4. Determinants of the Difference Between Brachial Cuff and Invasive Aortic Systolic Blood Pressures (Sensitivity Analyses With Body Mass Index or Body Surface Area Instead of Weight) eTable 5. Comparison of Invasive and Non-Invasive Blood Pressures According to Tertiles of Height eTable 6. Comparison of Invasive and Non-Invasive Blood Pressures According to Tertile of Height and Sex eTable 7. Comparison of Invasive and Non-Invasive Blood Pressures According to Age Groups and Sex eFigure 1. Study Flowchart eFigure 2. Modified Bland-Altman Plots According to Sex and Type of Non-Invasive Systolic Blood Pressure Measurement eFigure 3. Mean Differences Between Invasive (Brachial and Aortic) and Non-Invasive (Brachial Cuff, Type I Central and Type II Central) Diastolic Blood Pressure According to Sex and Tertiles of Height [file jamanetwopen-e2215513-s001.pdf]

Supplementary Online Content

Abbaoui Y, Fortier C, Desbiens LC, et al. Accuracy difference of noninvasive blood pressure measurements by sex and height. *JAMA Netw Open*. 2022;5(6):e2215513. doi:10.1001/jamanetworkopen.2022.15513

eMethods. Supplemental Methods

eTable 1. Comparison of Invasive and Non-Invasive Blood Pressures in Females and Males in the Subset of Participants With Available Invasive Brachial Blood Pressure

eTable 2. Difference Between Invasive Central Aortic SBP and Non-Invasive Brachial Cuff SBP According to Sex and Various Procedural Factors

eTable 3. Determinants of the Difference Between Brachial Cuff and Invasive Aortic Systolic Blood Pressures

eTable 4. Determinants of the Difference Between Brachial Cuff and Invasive Aortic Systolic Blood Pressures (Sensitivity Analyses With Body Mass Index or Body Surface Area Instead of Weight)

eTable 5. Comparison of Invasive and Non-Invasive Blood Pressures According to Tertiles of Height

eTable 6. Comparison of Invasive and Non-Invasive Blood Pressures According to Tertile of Height and Sex

eTable 7. Comparison of Invasive and Non-Invasive Blood Pressures According to Age Groups and Sex

eFigure 1. Study Flowchart

eFigure 2. Modified Bland-Altman Plots According to Sex and Type of Non-Invasive Systolic Blood Pressure Measurement

eFigure 3. Mean Differences Between Invasive (Brachial and Aortic) and Non-Invasive (Brachial Cuff, Type I Central and Type II Central) Diastolic Blood Pressure According to Sex and Tertiles of Height

This supplementary material has been provided by the authors to give readers additional information about their work.

© 2022 Abbaoui Y et al. *JAMA Network Open.*

eMethods. Supplemental Methods

Invasive BP measures

During all measurements, patients remained in a supine position with instructions to breathe normally and not to speak. After catheter insertion, patients were administered a mixture of intraarterial vasodilators (0.5-1 mg of verapamil with 300-600 mcg nitroglycerin), and instructions were given to avoid administering any vasoactive or contrast agent during and at least 5 minutes prior to all study recordings unless clinically indicated. Invasive BP measurements were performed at the end of the clinical procedure. Fluid-filled catheters (5F or 6F; 100 cm) were used, which were connected using three taps and connectors to a Xper Flex Cardio Physiomonitoring System (Philips, Amsterdam, Netherlands) with the manifold position maintained at heart level. At the end of the coronary angiography procedure, intraarterial BPs were measured after pulling back the catheters into the aorta to within 3 cm of the aortic valve, (invasive aortic BP) with correct positioning confirmed by fluoroscopy. Bubbles were removed by aspirating blood before flushing 10 mL of 0.9% NaCl solution into the catheter. Before each measurement, the monitoring system was zeroed and calibrated. The damping coefficient and frequency response of the system were assessed to be >0.3 and >18 Hz respectively. Visual confirmation for the correct appearance of the waveform on the monitor was performed prior to all measurements. When possible, additional recordings were taken after pulling back the catheter mid-humerus to the brachial artery (invasive brachial BP). Visual re-assessment of the dampening of the curve was done and if necessary, flushing, calibration and zero were repeated. All invasive BP measurements were digitally recorded during 20 seconds and for every heartbeat, then averaged.

Non-invasive BP measures

The Mobil-O-Graph NG device (I.E.M., Stolberg, Germany), a validated oscillometric device [1,2], was used for all non-invasive brachial and central BP readings. Through pulse wave analysis and a generalized transfer function, this device first measures brachial cuff BP then re-inflates to capture the pulse waveform and derive non-invasive central BP. It simultaneously provides central BP calibrated with either SBP/diastolic BP (DBP) or with mean arterial pressure (MAP)/DBP. As such, the Mobil-O-Graph yields what is conventionally called Type I and Type II non-invasive central BP, respectively. It can also calculate indices of arterial stiffness and wave reflection such as the aortic pulse wave velocity (PWV), the augmentation index and reflection magnitude. Before angiography, the arm contralateral to the planned arterial access site was measured to determine the appropriate cuff size. Then, the Mobil-O-Graph cuff was placed on this arm and the patient comfortably installed on the angiography table. Prior to the beginning of the cardiac catheterization, correct positioning and signal capture were tested. At the end of the angiography procedure, recordings of non-invasive brachial and central BPs were made simultaneously to the invasive aortic measurement, timed to coincide with the cuff deflation.

1. Weber T, Wassertheurer S, Rammer M, et al. Validation of a brachial cuff-based method for estimating central systolic blood pressure. *Hypertension.* 2011;58(5):825-832.

2. Jones CR, Taylor K, Chowienczyk P, Poston L, Shennan AH. A validation of the Mobil O Graph (version 12) ambulatory blood pressure monitor. *Blood pressure monitoring.* 2000;5(4):233-238.

© 2022 Abbaoui Y et al. *JAMA Network Open.*

eTable 1: Comparison of invasive and non-invasive blood pressures in females and males in the subset of participants with available invasive brachial blood pressure.

BP components (mmHg)	Females (n=80)	Males (n=223)	p-value
Brachial cuff SBP	123.4 (17.4)	123.1 (15.5)	0.89
Invasive brachial SBP	136.2 (20.8)	130.8 (20.3)	0.044
Invasive aortic SBP	129.9 (21.7)	123.9 (20.1)	0.024
Invasive SBP amplification	6.3 (10.3)	6.8 (9.1)	0.65
**Difference from Intra-Aortic SBP**			
Brachial cuff SBP	-6.5 (13.3)	-0.8 (11.3)	<0.001
Type I non-invasive central SBP	-15.8 (14.0)	-9.5 (12.7)	<0.001
Type II non-invasive central SBP	0.4 (17.6)	8.2 (14.6)	<0.001
Brachial cuff DBP	73.5 (11.2)	77.1 (10.0)	0.007
Invasive brachial DBP	68.6 (11.0)	68.6 (10.0)	0.99
Invasive aortic DBP	69.6 (11.1)	68.7 (9.8)	0.53
**Difference from Intra-Aortic DBP**			
Brachial cuff DBP	3.9 (9.3)	8.4 (7.6)	<0.001
Type I non-invasive central DBP	5.1 (9.3)	9.6 (7.9)	<0.001
Type II non-invasive central DBP	5.5 (10.8)	10.3 (8.3)	<0.001
Pulse pressure amplification	1.14 (0.19)	1.15 (0.22)	0.63

Results are mean (SD). P-values for sex comparisons were obtained with t-tests. Mean differences represent the difference between the non-invasive blood pressure and the intraarterial invasive aortic blood pressure. SBP, systolic blood pressure; DBP, diastolic blood pressure. Type I non-invasive central BP is obtained through calibration with brachial cuff SBP and DBP. Type II non-invasive central BP is obtained through calibration with brachial cuff mean BP and DBP. All values are expressed as mmHg.

© 2022 Abbaoui Y et al. *JAMA Network Open.*

eTable 2: Difference between invasive central aortic SBP and non-invasive brachial cuff SBP according to sex and various procedural factors.

	Females		Males		
	n	Accuracy	n	Accuracy	p-values
**Catheter gauge**					
5 French	77	-6.1 (11.9)	104	1.2 (11.6)	<0.001
6 French	68	-6.9 (12.4)	251	-1.0 (11.7)	<0.001
p-values		0.70		0.10	
**Vasoactive drugs within 5 minutes**					
Yes	45	-5.8 (11.0)	69	0.3 (11.3)	0.005
No	100	-6.8 (12.6)	286	-0.5 (11.9)	<0.001
p-values		0.65		0.60	
**Measures performed by primary study cardiologist**					
Yes	77	-4.7 (11.3)	186	-1.1 (10.8)	0.02
No	68	-8.0 (12.6)	169	0.4 (12.5)	<0.001
p-values		0.10		0.24	
**Mobil-o-Graph signal quality**					
1 (best)	81	-6.9 (12.7)	182	0.5 (11.7)	<0.001
2 or 3 [a]	64	-5.9 (11.1)	173	-1.1 (11.7)	0.005
p-values		0.63		0.22	

Accuracy represents the mean difference between the non-invasive brachial cuff and the invasive central (aortic) systolic blood pressure and is expressed with standard deviations.
[a] 9 females and 8 males had signal quality 3.

© 2022 Abbaoui Y et al. *JAMA Network Open.*

eTable 3: Determinants of the difference between brachial cuff and invasive aortic systolic blood pressures.

Covariables	B coefficient	95% CI	p-value
Sex	3.58	0.27 to 6.89	0.034
Age (year)	-0.35	-0.68 to -0.01	0.042
Height (cm)	0.27	0.11 to 0.43	0.001
Weight (kg)	-0.04	-0.10 to 0.03	0.27
Brachial cuff DBP (mmHg)	-0.12	-0.23 to 0.00	0.051
Brachial cuff pulse pressure (mmHg)	0.11	-0.01 to -0.24	0.063
Heart rate (bpm)	0.05	-0.04 to 0.14	0.30
Augmentation index @ 75 bpm	-0.04	-0.13 to 0.05	0.42
Aortic pulse wave velocity (m/s)	0.95	-1.0 to 2.89	0.34
eGFR (mL/min/1.73m^2)	-0.05	-0.11 to 0.02	0.20
Active smoking status	-0.12	-2.71 to 2.48	0.93
Type 2 diabetes	-0.76	-3.12 to 1.64	0.53
Presence of ≥ 1 diseased vessel	-1.00	-3.36 to 1.37	0.41
Use of antihypertensive drugs	-0;67	-3.39 to 2.05	0.63
Use of aspirin	-0.14	-2.64 to 2.36	0.91
Use of statin	0.73	-1.64 to 3.11	0.54

Multivariate linear regression analysis. eGFR, glomerular filtration rate estimated using the CKD-EPI formula; DBP, diastolic blood pressure; CI, confidence interval.

eTable 4: Determinants of the difference between brachial cuff and invasive aortic systolic blood pressures (sensitivity analyses with body mass index or body surface area instead of weight).

Covariables	B coefficient	95% CI	p-value	B coefficient	95% CI	p-value
Sex	3.73	0.42 to 7.03	0.027	3.74	0.44 to 7.04	0.026
Age (year)	-0.36	-0.69 to -0.03	0.034	-0.36	-0.69 to -0.03	0.035
Height (cm)	0.22	0.07 to 0.38	0.004	0.29	0.10 to 0.49	0.003
Weight (kg)	-	-	-	-	-	-
Body mass index (kg/m^2)	-0.12	-0.31 to 0.08	0.23	-	-	-
Body surface area (m^2)	-	-	-	-3.87	-10.85 to 3.12	0.28
Brachial cuff DBP (mmHg)	-0.12	-0.24 to -0.00	0.045	-0.12	-0.24 to -0.00	0.046
Brachial cuff pulse pressure (mmHg)	0.11	-0.01 to 0.23	0.065	0.11	-0.01 to 0.23	0.066
Heart rate (bpm)	0.05	-0.05 to 0.14	0.30	0.05	-0.04 to 0.14	0.30
Augmentation index @ 75 bpm	-0.04	-0.13 to 0.05	0.43	-0.04	-0.13 to 0.05	0.42
Aortic pulse wave velocity (m/s)	0.97	-0.97 to 2.92	0.33	0.97	-0.97 to 2.91	0.33
eGFR (mL/min/1.73m^2)	-0.04	-0.11 to 0.03	0.21	-0.04	-0.11 to 0.03	0.21
Active smoking status	-0.07	-2.67 to 2.52	0.96	-0.05	-2.64 to 2.55	0.97
Type 2 diabetes	-0.81	-3.21 to 1.59	0.51	-0.82	-3.22 to 1.58	0.50
Presence of ≥ 1 diseased vessel	-0.91	-3.27 to 1.45	0.45	-0.91	-3.27 to 1.45	0.45
Use of antihypertensive drugs	-0.62	-3.34 to 2.09	0.65	-0.62	-3.34 to 2.09	0.65
Use of aspirin	-0.11	-2.61 to 2.39	0.93	-0.11	-2.60 to 2.39	0.93
Use of statin	0.78	-1.59 to 3.15	0.52	0.78	-1.59 to 3.15	0.52

Multivariate linear regression analysis. eGFR, glomerular filtration rate estimated using the CKD-EPI formula; DBP, diastolic blood pressure; CI, confidence interval.

© 2022 Abbaoui Y et al. *JAMA Network Open.*

eTable 5: Comparison of invasive and non-invasive blood pressures according to tertiles of height.

BP components (mmHg)	Height < 167 cm (n=158)	Height 167 to 175 cm (n=189)	Height ≥ 176 cm (n=153)	p-value
**SBP measurements**				
Invasive Aortic	132.9 (21.4)	125.8 (20.2)	120.9 (18.9)	<0.001
Invasive Brachial *	136.2 (19.7)	133.0 (20.9)	127.5 (20.3)	0.013
Brachial Cuff	126.4 (18.0)	123.8 (16.6)	123.1 (15.7)	0.20
Type I non-invasive central	115.5 (16.9)	115.5 (16.1)	115.1 (15.5)	0.96
Type II non-invasive central	133.9 (20.0)	132.0 (17.4)	132.3 (17.4)	0.60
**Difference from Invasive SBP**				
Brachial cuff (vs invasive brachial) [a]	-11.6 (14.3)	-10.9 (12.5)	-5.9 (13.2)	0.012
Brachial cuff (vs invasive aortic)	-6.6 (11.5)	-1.9 (12.0)	2.3 (11.3)	<0.001
Type I central (vs invasive aortic)	-17.4 (13.2)	-10.3 (12.8)	-6.3 (13.4)	<0.001
Type II central (vs invasive aortic)	0.9 (14.8)	6.3 (13.4)	11.1 (15.2)	<0.001

[a] Data available in 89, 118 and 96 participants, respectively
Mean differences represent the difference between the non-invasive blood pressure and the intraarterial invasive aortic blood pressure and are expressed with standard deviations. SBP, systolic blood pressure. Type I non-invasive central BP is obtained through calibration with brachial cuff SBP and DBP. Type II non-invasive central BP is obtained through calibration with brachial cuff mean BP and DBP. P-values are calculated one-way ANOVA with Bonferroni corrections for post-hoc analyses.

© 2022 Abbaoui Y et al. *JAMA Network Open.*

eTable 6: Comparison of invasive and non-invasive blood pressures according to tertiles of height and sex.

	Females		Males		
	n	Accuracy (mmHg)	n	Accuracy (mmHg)	p-values
**Brachial cuff SBP**					
Height < 167 cm	124	-7.0 (12.1)	34	-4.9 (9.0)	0.33
Height 167 to 175 cm	20	-2.9 (12.0)	169	-1.7 (12.0)	0.69
Height ≥ 176 cm	1	-6.0	152	2.3 (11.3)	0.46
p-values		0.15 [a]		<0.001	
**Type I Central SBP**					
Height < 167 cm	124	-18.0 (13.4)	34	-15.2 (12.6)	0.28
Height 167 to 175 cm	20	-13.4 (10.3)	169	-9.8 (13.0)	0.24
Height ≥ 176 cm	1	-8.0	152	-6.1 (12.6)	0.88
p-values		0.15 [a]		<0.001	
**Type II Central SBP**					
Height < 167 cm	124	-0.0 (15.5)	34	4.4 (11.3)	0.12
Height 167 to 175 cm	20	4.0 (14.0)	169	6.6 (13.3)	0.41
Height ≥ 176 cm	1	1.0	152	11.2 (15.2)	0.50
p-values		0.28 [a]		0.003	

[a] p-values refers to comparison between first and second tertiles of height, as n=1 in the highest tertile.
Accuracy represents the mean differences (with standard deviation) between each non-invasive blood pressure measurement and the intraarterial invasive aortic blood pressure. P-values compare females to males and are calculated using one-way ANOVA with Bonferroni corrections for post-hoc analyses.

© 2022 Abbaoui Y et al. *JAMA Network Open.*

eTable 7: Comparison of invasive and non-invasive blood pressures according to age groups and sex.

	Overall		Females		Males		
	n	Accuracy (mmHg)	n	Accuracy (mmHg)	n	Accuracy (mmHg)	p-values
**Brachial cuff SBP**							
Age 40-60 years	138	0.3 (12.5)	41	-5.9 (10.9)	97	2.9 (12.2)	<0.001
Age 60-80 years	329	-2.7 (11.8)	92	-5.6 (12.2)	277	-1.6 (11.4)	0.005
Age 80+ years	33	-5.7 (12.8)	12	-12.8 (12.4)	21	-0.5 (10.1)	0.001
p-values		0.011		0.044		0.007	
**Type I Central SBP**							
Age 40-60 years	138	-8.2 (12.8)	41	-15.6 (10.6)	97	-5.1 (12.5)	<0.001
Age 60-80 years	329	-12.0 (13.4)	92	-16.8 (13.3)	277	-10.2 (13.0)	<0.001
Age 80+ years	33	5.8 (14.9)	12	-27.2 (15.9)	21	-9.6 (13.6)	0.002
p-values		0.002		0.020		0.004	
**Type II Central SBP**							
Age 40-60 years	138	7.3 (15.2)	41	-0.3 (12.5)	97	10.5 (15.1)	<0.001
Age 60-80 years	329	5.8 (14.9)	92	1.5 (16.8)	277	7.5 (13.8)	<0001
Age 80+ years	33	3.8 (14.1)	12	-3.7 (11.5)	21	8.1 (13.8)	0.019
p-values		0.41		0.50		0.21	

Accuracy represents the mean differences (with standard deviation) between each non-invasive blood pressure measurement and the intraarterial invasive aortic blood pressure. P-values compare females to males and are calculated using one-way ANOVA with Bonferroni corrections for post-hoc analyses.

© 2022 Abbaoui Y et al. *JAMA Network Open.*

eFigure 1: Study flowchart

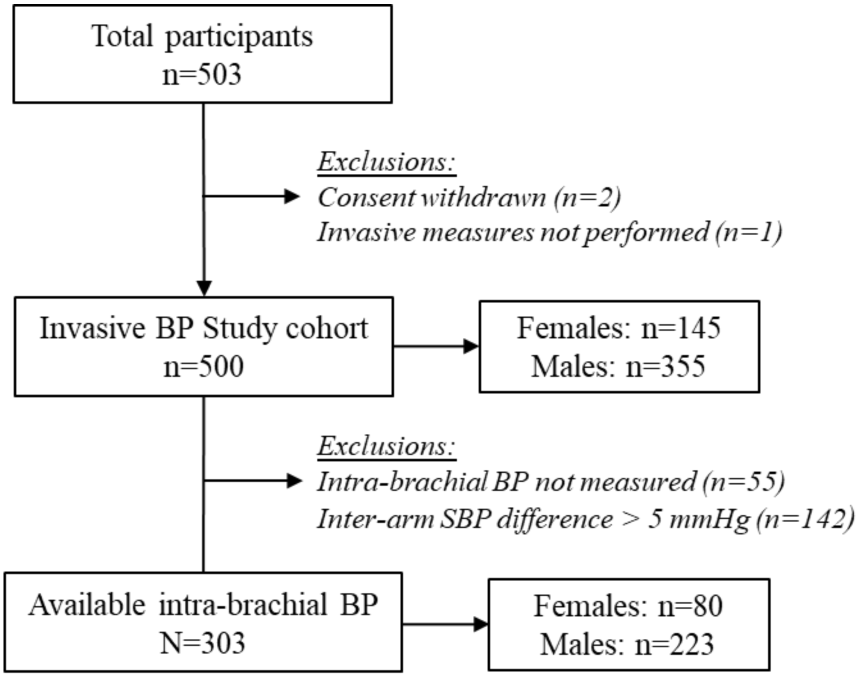

BP, Blood pressure; SBP, Systolic blood pressure.

© 2022 Abbaoui Y et al. *JAMA Network Open.*

eFigure 2: Modified Bland-Altman plots according to sex and type of non-invasive systolic blood pressure measurement.

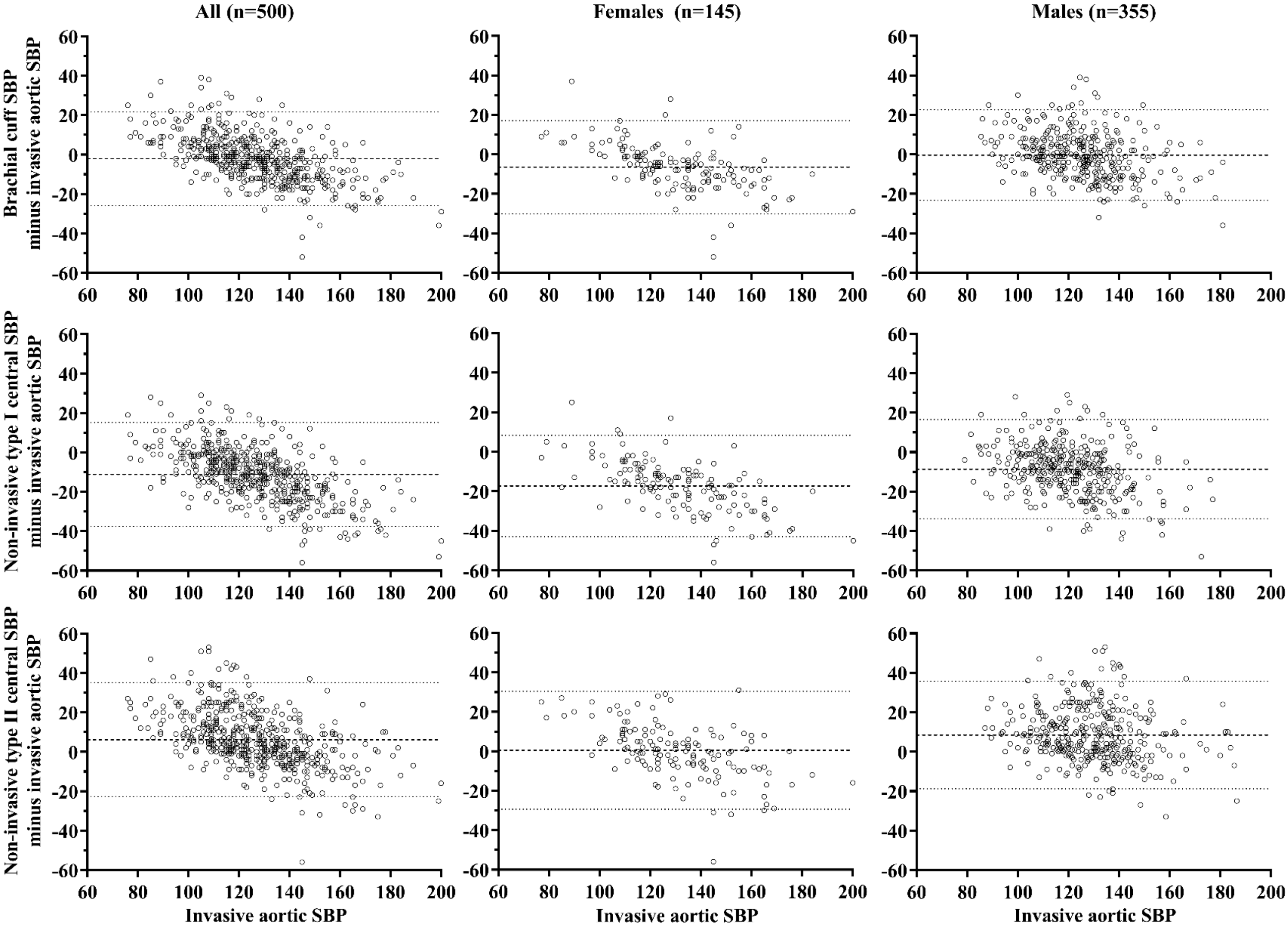

Modified Bland-Altman plots show the reference measurement (invasive aortic SBP) on the X axis instead of the mean of both measurements. Left to right panels show in order the data for the overall cohort, females and males. Top to bottom panels show data for brachial cuff SBP, non-invasive type I central SBP and non-invasive type II central SBP. All values are in mmHg. Dashed lines and dotted lines represent the mean differences (bias) and limits of agreement (bias ± 2 standard deviations).

© 2022 Abbaoui Y et al. *JAMA Network Open.*

eFigure 3: Mean differences between invasive (brachial and aortic) and non-invasive (brachial cuff, Type I central and Type II central) diastolic blood pressure according to sex and tertiles of height.

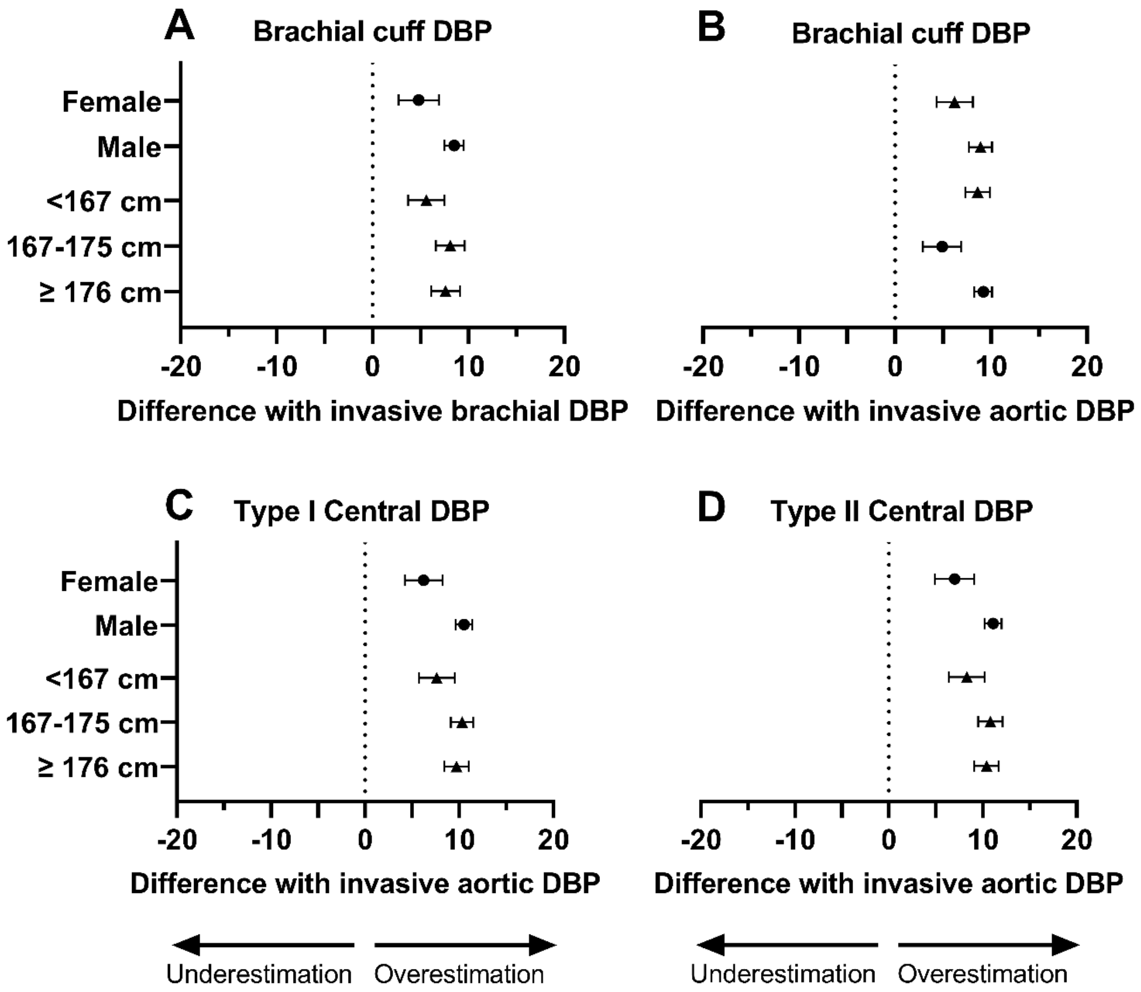

Panel A: Differences between brachial cuff DBP and intra-brachial DBP; Panel B: between brachial cuff DBP and intra-aortic DBP; Panel C: between Type I central DBP and intra-aortic DBP; Panel D: between Type II central DBP and intra-aortic DBP. Circles and triangles represent data according to sex and tertiles of height, respectively, and brackets show the 95% confidence intervals. Dotted line represents the point where neither overestimation nor underestimation occur, indicative of high accuracy.

© 2022 Abbaoui Y et al. *JAMA Network Open.*
